# Movement variability in Pilates: a scoping review

**DOI:** 10.3389/fpsyg.2023.1195055

**Published:** 2023-09-15

**Authors:** Mário José Pereira, Gonçalo Dias, Rui Mendes, Fernando Martins, Ricardo Gomes, Maria António Castro, Vasco Vaz

**Affiliations:** ^1^Faculdade de Ciências do Desporto e Educação Física, Universidade de Coimbra, Coimbra, Portugal; ^2^Escola Superior de Educação de Coimbra, Instituto Politécnico de Coimbra, Coimbra, Portugal; ^3^Laboratório RoboCorp, IIA, Instituto Politécnico de Coimbra, Coimbra, Portugal; ^4^CIDAF (UID/DTP/04213/2020), Universidade de Coimbra, Coimbra, Portugal; ^5^ESEC-UNICID-ASSERT, Instituto Politécnico de Coimbra, Coimbra, Portugal; ^6^Instituto de Telecomunicações, Delegação da Covilhã, Covilhã, Portugal; ^7^Escola Superior de Saúde, Instituto Politécnico de Leiria, Leiria, Portugal

**Keywords:** movement science, motor learning and control, Sport Sciences, non-linear analysis, Pilates

## Abstract

**Objective:**

This scoping review aimed to identify studies that analyzed movement variability in Pilates. Following a systematic approach to mapping evidence on this topic would highlight concepts, theories, sources, and knowledge gaps in this area.

**Methods:**

This review used the Preferred Reporting Items for Systematic Reviews and Meta-Analyses extension for Scoping Reviews (PRISMA-ScR) criteria for the selection, reading, and analysis of studies in this area. We searched five literature databases (Web of Science, SCOPUS, library catalog of the Faculty of Sport and Physical Education of the University of Coimbra—EBSCO Discovery Services, MEDLINE, and Google Scholar). Eligible articles contained the word “Pilates,” and the human movement variability was analyzed. Any type of study (except reviews) could be eligible and must have been published between 1 January 2002 and 30 November 2022, in Portuguese, Spanish, French, or English.

**Results:**

Our search identified five eligible entries. Only one study used the Pilates method in its intervention, pointing to a more significant variability of hip–knee coordination, suggesting more diversified coordination patterns, and maintaining the variability of the angular position of the joint.

**Conclusion:**

Very few studies have examined movement variability in Pilates, and only one applied an ecological framework.

## 1. Introduction

Variability can be described as the normal variation in movement during the repetition of a given task (Cowin et al., 2022). Stergiou and Decker (2011) argued that human movement is too complex to be effectively analyzed using only quantitative linear techniques or approaches closer to cognitive theories (Schmidt, 1975), such as averages, standard deviations, and coefficients of variation commonly employed in linear statistics. Instead, a non-linear analysis approach considers human movement within the framework of biological and naturalistic rules, supporting the use of data established in time series and analyzed through the lens of non-linear tools such as sample entropy (SampEn), fractal analysis (DFA), and Lyapunov exponent (PyE) (Stergiou and Decker, 2011; Hadamus et al., 2022).

van Emmerik and van Wegen (2000) indicated that the traditional perspective in biology and movement science is limited in its ability to deeply analyze the impact of noise and variability on performance and various pathologies. Embracing variability introduces new perspectives to the study of motor control systems by including new elements, such as periodicity, self-similarity, and long-range correlation (Haworth, 2008). Araújo et al. (2009) suggested that employing the concepts of ecological dynamics increases our understanding of phenomena as they unfold in the context of the subject–environment relationship. The ecological perspective seeks to understand and explain the phenomenon in its context, encompassing the greatest number of factors that may influence it. The expected outcome is a “constraints-based framework” that maintains experimental rigor even when conducted in field settings (Davids et al., 2003). Aligned with this view, Newell (1986) conceptualized skill acquisition as an evolving ecological practice, where athletes must cope with complexity, integrated and non-linear reciprocity of the practitioner's organism, the task itself, and the environmental subsystems.

The philosophy of Pilates emphasizes individualization, attending to human differences, and variability of movements. In Pilates, the goal is not to achieve a specific standard of performance, but rather to improve the individual's quality of movement. This approach is based on the belief that a dynamic and adaptable motor system is more responsive, healthy, and functional than a static one (Stergiou and Decker, 2011). Such an analysis can facilitate understanding of the mechanisms underlying learning and development, and identify specific characteristics of Pilates movements that can contribute to their effectiveness, especially concerning the variability of the Pilates method.

Based on the above analysis, this scoping review aimed to identify studies that analyzed movement variability in Pilates. Following a systematic approach to mapping evidence on a topic, we sought to highlight concepts, theories, sources, and knowledge gaps in the research field.

## 2. Material and methods

### 2.1. Study design and methodology

This scoping review aimed to understand the role of variability in Pilates using an ecological systems approach to human motor control. The implications of knowledge extend to various fields and some promising results in medicine (Silva da Rocha et al., 2022). By adopting this perspective, a new dimension in Pilates research can be explored, potentially yielding valuable insights for future interventions and research directions.

So, it is important to establish the difference between the variability, as a measure of linear analysis calculated based on the central measure (e.g., standard deviation or the coefficient of variation), and the variability included in the non-linear dynamic analysis (NDA), working with time series to study dynamical systems (Cavanaugh et al., 2005).

### 2.2. Search strategy

**Figure 1:**
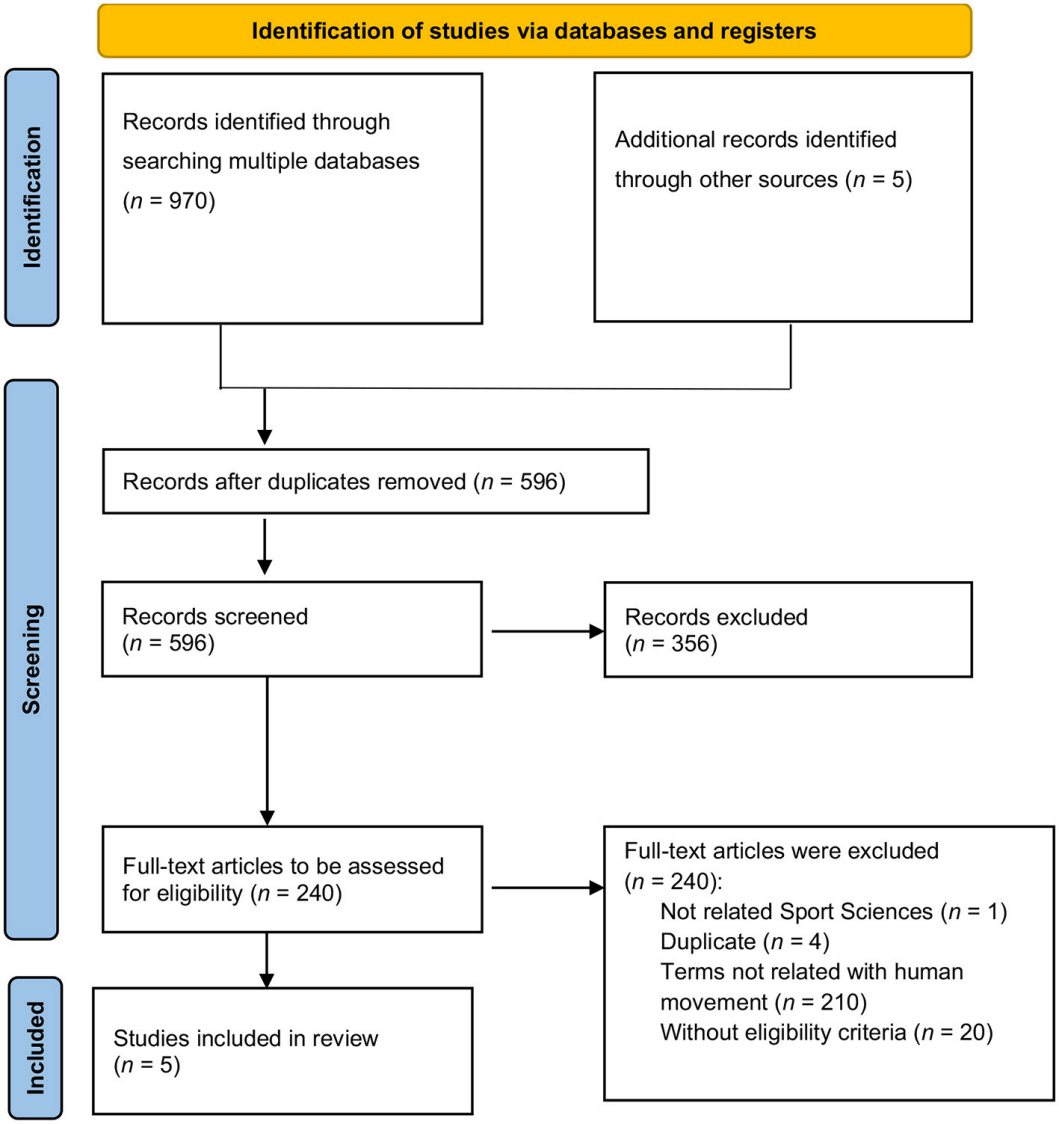
PRISMA 2020 flow diagram for new systematic reviews, which included searches of databases and registers only (adapted from Page et al., 2021).

The Preferred Reporting Items for Systematic Reviews and Meta-Analyses extension for Scoping Reviews (PRISMA-ScR) (Tricco et al., 2018) criteria were used for the selection, reading, and analysis of studies in the selected research area. This extension of the original PRISMA statement adapts the objectives and methodological approach; the checklist includes 20 items plus 2 optional items and provides guidance on the reporting of scoping reviews. The search was performed on 1 December 2022 in five databases (Figure 1): (i) Web of Science (Core Collection), (ii) SCOPUS, (iii) Search directory of the library catalog of the Faculty of Sport and Physical Education of the University of Coimbra—EBSCO Discovery Services, (iv) MEDLINE, and (v) Google Scholar.

The final version of the protocol was registered on the Open Science Framework website, as an open model, on 22 November 2022 (https://osf.io/ktqp8/). The number and selection of databases in this study were consistent with previous research on Pilates to ensure comprehensive coverage of relevant articles. The search strategy employed a combination of the keywords “Pilates” or “Pilates-based” in the title or abstract, in conjunction with “variability”, or “nonlinear measures” or “nonlinear analysis” or “ecological” or “dynamic”. In the SCOPUS database, the search encompassed these words in the “keywords” field. For the Web of Science database, the search was limited to the abstract section, and in Google Scholar, the search was focused on the title field.

### 2.3. Eligibility criteria

For this scoping review, four criteria were established for article inclusion: (i) articles whose title contained the word “Pilates” and Pilates was one of the dependent variables; (ii) the analysis of the results included the variability of human movement; (iii) the articles were published between 1 January 2002 and 30 November 2022; and (iv) the articles were written in Portuguese, Spanish, French, or English.

Exclusion criteria included reviews and articles without full-text access. Limiting the exclusion criteria to these two factors aimed to cover the largest possible number of studies with these variables, given the predictable small number of studies in this area. For the same reason, the time window for article inclusion was from 2002 to 2022 to increase the possibility of finding relevant literature and to allow the proper inclusion of these themes in Psychology and Sport Sciences. The selected languages were based on the authors' abilities to prevent translation biases. Furthermore, previous reviews (Pereira et al., 2022; Silva da Rocha et al., 2022) indicated that these databases and languages adequately represent the state of the art in the field of Pilates.

The article selection process involved several steps completed independently by authors (M.P., R.M.), followed by a comparison and discussion of their eligibility. In case of differences, a third author (G.D.) was consulted to analyze and produce a final decision. The following procedures were adopted: (i) searching the databases for studies using the specified descriptors; (ii) excluding duplicate articles; (iii) reading the titles and abstracts; and (iv) reading and critically evaluating the articles (see Figure 1). Gray literature, such as communications in congresses, opinion articles, and other non-scientific sources, was included when found and eligible.

## 3. Results

From the five databases analyzed, a total of five entries were eligible for inclusion in this study after comprehensive reading and analysis of the articles. A structured summary of the included studies is presented in Table 1.

**Table 1:** Structured summary of the studies included in the analysis.

**References**	**Title**	**Sample**	**Study aim**	**Intervention**	**Comparison**	**Outcomes**	**Variability's approach**
Claeys et al. (2011)	*Decreased variability in postural control strategies in young people with non-specific low back pain is associated with altered proprioceptive reweighting*	Young individuals with non-specific low back pain (NSLBP) (x = 18.5 years, sd = 0.5) and control/healthy group (x = 19.6 years, sd = 1.6)	Investigate if young people choose the optimal postural control strategy according to their postural condition; investigate if NSLBP influences the variability in proprioceptive postural control strategies	No intervention	Control group	Means of muscle vibration on triceps surae and lumbar multifidus muscles; root mean square and mean displacements of the center of pressure; relative proprioceptive weighting (RPW, ratio of ankle vs. back muscles proprioceptive inputs)	Young healthy people have the ability to choose the optimal multi-segmental postural control strategy according to their postural condition. In contrast, young people with mild NSLBP show reduced variability in proprioceptive postural control strategies due to decreased proprioceptive reweighting capacity. This loss of variability in strategy selection is associated with decreased postural robustness. Decrease in variability is associated with postural control inefficiency.
Foreman et al. (2011)	*Testing balance and fall risk in persons with Parkinson's disease, an argument for ecologically valid testing*	Parkinson's disease (PD) patients, fallers (x = 70.95 sd = 11.41) and non-fallers (x = 66.64 sd = 10.05)	Examine ecologically valid testing of postural instability and fall risk in persons with Parkinson's disease	No intervention. Test after 12 h without PD medication and retest 1–1.5 h after medication	ON–OFF PD medication	The responsiveness and predictive validity of the Functional Gait Assessment (FGA), the Pull test, and the Timed up and Go (TUG)	To accurately identify fallers, clinicians should test persons with PD in ecologically relevant conditions and tasks.
Roh et al. (2016)	*Effects of modified Pilates on variability of inter-joint coordination during walking in the elderly*	Twenty elderly participants. Two groups: Trained (10) = 67.6 (2.4) years, Control (10) = 68.1 (2.9) years	Examine the effects of an 8-week modified Pilates program on inter-joint coordination variability during walking	Effects of an 8-week modified Pilates program on the variability of inter-joint coordination during walking	Trained/control group	Angle and angular velocity of lower extremity joints; inter-joint coordination variability through the deviation phase (DP)	The exercise group showed greater hip–knee coordination variability, whereas joint variability remained unchanged, due to the development of deep muscles through Pilates, which helped to improve pelvis/hip flexibility. This suggests that more diverse coordination patterns are obtained, maintaining the variability of joint angular position. Variability is built in when building good, healthy motor patterns.
Sanders (2007)	*Kinematics, coordination, variability, and biological noise in the prone flutter kick at different levels of a “learn-to-swim” program*	Nine children from three levels of a “learn-to-swim” program, and 10 skilled swimmers were video-recorded doing prone flutter kicking	Establish the movement patterns common to flutter kicking of skilled swimmers and compare them to the movement patterns of swimmers at different levels of a “learn-to-swim” program	No intervention	Skilled group Learning group	Kinematics, including joint angular motion and coordination of joint actions.	There was strong evidence to suggest that skilled performance in flutter kicking is characterized by sequencing joint actions to produce a single sinusoidal body wave moving caudally without decreasing and preferably by increasing velocity, low biological noise, and small variability. Variability is presented as a part of motor competency.
Seay et al. (2011)	*Low back pain status affects pelvis-trunk coordination and variability during walking and running*	Three groups of recreational runners, aged 18–40, who ran at least 20 km/week.	Compare pelvis–trunk coordination and coordination variability over a range of walking and running speeds between three groups of runners	No intervention	LBP group (n = 14) Resolved (RES) group (n = 14) Remaining group (CTR group, n = 14)	Means and standard deviations were reported for continuous relative phase (CRP) and CRP variability for all groups at all seven treadmill speeds.	The LBP showed decreased coordination variability, and this finding contributes to a growing body of literature that suggests that decreased coordination variability during certain tasks may be indicative of pathology. In this study, variability is a trait associated with postural efficacy and can help to avoid low back pain.

The studies collected and identified in our search were not fully compatible with the variability approach using NDA and the ecological approach of Pilates' practice. Claeys et al. (2011) searched the variability of postural control strategy between young subjects with non-specific low back pain (NSLBP) and young healthy subjects. The authors associated the reduction in variability with the decrease in proprioceptive reweighting capacity and as a result of the decrease in postural robustness. Foreman et al. (2011) compared the risk of falling in Parkinson's disease patients with and without medication and concluded that assessment in an ecological environment and tasks should be used to produce more accurate data on clinical analysis. In another study, Roh et al. (2016) studied the influence of Pilates intervention on gait in the elderly. The results showed increased hip–knee coordinative variability, suggesting more diverse coordination patterns while maintaining the variability of the joint angular position. An observational study (Sanders, 2007) revealed better joint action when there was increasing velocity, low biological noise, and small variability in flutter kicking of skilled swimmers. Finally, Seay et al. (2011) associated the decreased coordination variability during specific tasks in a low back pain group with a decreased coordination variability, which should be indicative of pathology.

## 4. Discussion

According to the eligibility requirements defined in this research, only five studies found in the databases met the inclusion criteria. In four studies, variability was addressed as an environmental factor—a natural and necessary condition in human movement to achieve good posture or learn a motor skill. Only one study (Roh et al., 2016) specifically used the Pilates method in their intervention. It is also noteworthy that none of the studies used NDA. Based on our review, there is a lack of information regarding the influence of variability on the learning and performance of movements and skills in Pilates. Although the search found numerous studies using the terms “variability” or “ecological,” many of them were not directly relevant to the specific context of this research. Indeed, the concept “variability” is also a measure of linear analysis (e.g., standard deviation or confidence intervals), which explains the gap, distance, and fluctuation of data about a central tendency, usually indexed to the cognitive theories.

The advantages of Pilates are well documented in the literature (Mazzarino et al., 2015, 2022; Franks et al., 2023). However, the analysis of these advantages could be further enhanced by considering the variability studied through NDA, as mentioned earlier. NDA has demonstrated potential in various areas, such as gait (Suarez-Iglesias et al., 2019; Chen et al., 2021), static and dynamic balance with a pressure platform (Martinez-Sanchez et al., 2020; Fernandez-Rodriguez et al., 2022), and postural analysis (Cavanaugh et al., 2005). For example, Tsigkanos et al. (2021) associated health conditions with greater movement variability in kinematic behavior, during gait analysis, whereas Amirpourabasi et al. (2022) highlighted the power of non-linear analysis in gait analysis, contributing to the development of reference values for estimating the risk of falling. Furthermore, Kedziorek and Błazkiewicz (2020) concluded that reductions in postural regularity can be related to aging, pathology, or injury, impacting the adaptive capabilities of the movement system and how predictability changes under different task constraints. In a scoping review, Strongman and Morrison (2020) highlighted the usefulness of the NDA for the effective diagnosis and assessment of injuries as a promising area of research. Collectively, these studies have implicated the role of variability in learning and performance, from both a performance and a health standpoint.

Non-linear measures could provide new insights and powerful analysis in the field of Pilates, particularly in relation to health. Several authors (Stins et al., 2009; Rigoldi et al., 2013; Quek et al., 2014; Sempere-Rubio et al., 2018; Sun et al., 2019) have suggested that various diseases, injuries, or dysfunctions lead to decreased entropy values in populations. This can result in a loss of quality of postural control, decreased autonomy, and reduced interaction with the environment.

Conversely, in terms of performance, non-linear measures have shown higher values of entropy in the automated movements of dancers and gymnasts when compared with non-athletes (Stins et al., 2009; Muelas Perez et al., 2014).

In summary, numerous studies have identified various benefits of Pilates for their practitioners (Yook et al., 2022). The influence of Pilates on posture and movement quality due to flexibility or strength is well documented (Xu et al., 2023). However, the ecological dynamics perspective supported by NDA must be used in Pilates studies as variability is intrinsic to all biological systems. For instance, according to Harbourne and Stergiou's model (Harbourne and Stergiou), efficient and mature motor skills are related to healthy states. The model proposes that movement variability has a deterministic structure leading to the adaptability of the system, its stimuli, and stresses. This implies that the healthy condition of a biological system presents chaotic values in its time series. Overly ordered time series indicate excessive system predictability, whereas highly disturbed time series reflect an unpredictable system. Consequently, the motor system becomes more predictable, rigid, and mechanized when it exhibits reduced variability, which may have implications for health conditions (Harbourne and Stergiou, 2009).

## 5. Conclusion

This scoping review found only a single study on variability from an ecological perspective, indicating that further analysis is warranted on this topic.

## Author contributions

MP and GD conceptualized the study. MP, RM, and GD developed the methodology. MP, RM, MC, and VV validated the study. MP, FM, and GD performed data collection. MP, RM, RG, VV, and GD wrote the manuscript. MP, MC, FM, and GD edited the final version of the manuscript. RM: performing data collection. All authors have read and agreed to the published version of the manuscript.
